# Bodies adapt orientation-independent face representations

**DOI:** 10.3389/fpsyg.2013.00413

**Published:** 2013-07-11

**Authors:** Ellyanna Kessler, Shawn A. Walls, Avniel S. Ghuman

**Affiliations:** ^1^Department of Neurological Surgery, University of PittsburghPittsburgh, PA, USA; ^2^Department of Neurological Surgery, Center for the Neural Basis of Cognition, University of PittsburghPittsburgh, PA, USA

**Keywords:** face perception, body perception, perceptual adaptation, face inversion effect, body-face interaction, face adaptation, aftereffects, body inversion

## Abstract

Faces and bodies share a great number of semantic attributes, such as gender, emotional expressiveness, and identity. Recent studies demonstrate that bodies can activate and modulate face perception. However, the nature of the face representation that is activated by bodies remains unknown. In particular, face and body representations have previously been shown to have a degree of orientation specificity. Here we use body-face adaptation aftereffects to test whether bodies activate face representations in an orientation-dependent manner. Specifically, we used a two-by-two design to examine the magnitude of the body-face aftereffect using upright and inverted body adaptors and upright and inverted face targets. All four conditions showed significant body-face adaptation. We found neither a main effect of body orientation nor an interaction between body and face orientation. There was a main effect of target face orientation, with inverted target faces showing larger aftereffects than upright target faces, consistent with traditional face-face adaptation. Taken together, these results suggest that bodies adapt and activate a relatively orientation-independent representation of faces.

## Introduction

Faces and bodies provide a wealth of salient information that helps us navigate our social worlds and we employ specialized mechanisms to recognize and process these stimuli. Faces and bodies share useful properties: they co-occur at a high frequency and convey similar information about age, gender, and identity. Thus, information derived from the face and body can provide significant context to aid social perception. Recent studies demonstrate that perceptual representations of faces can be activated and modulated by viewing bodies without visible faces (Peelen and Downing, 2007; Brandman and Yovel, 2010; Ghuman et al., 2010; Brandman and Yovel, 2012; Schmalzl et al., 2012). However, little is known regarding the nature of the face representation activated by bodies. Here we use a recently described body-face adaptation aftereffect (Ghuman et al., 2010) to examine whether bodies activate faces according to the orientation of the body or in an orientation-independent manner.

Perceptual adaptation has been called the “psychologists' microelectrode” for its utility in carefully probing the nature of how stimuli are represented in the brain (Frisby, 1979). Perceptual adaptation is the process through which extended viewing of a stimulus produces an opposing aftereffect, such that a feature is more likely to be perceived as the opposite of that seen in the adapting stimulus. For instance, after viewing a line tilted to the right for several seconds, a vertical line is more likely to be perceived as tilting to the left (Gibson and Radner, 1937). When a stimulus is viewed for an extended period of time, the prolonged activation of neurons tuned to the properties of that stimulus elicits an adjustment of their response properties. This recalibration of the neurons' tuning is thought to underlie the measured perceptual adaptation aftereffects (Leopold et al., 2001; Clifford et al., 2007; Webster and MacLeod, 2011).

Perceptual adaptation has been reliably demonstrated to occur for a variety of visual properties, from basic aspects such as form and motion to higher-level qualities such as face identity (Leopold et al., 2001), gender (Webster et al., 2004), and expression (Fox and Barton, 2007). For instance, adapting to a male face results in an opposing aftereffect whereby subsequently viewed gender-neutral faces appear more feminine (Webster et al., 2004). Such effects are interpreted to reflect changes in the norm-based representation of the visual features and spatial relationships of faces, known as the “face space” (Leopold et al., 2001; Webster and MacLeod, 2011), which is used to determine face gender, identity, and expression. We have previously investigated how the “face space” is modulated by viewing bodies, finding that adapting to bodies without visible heads induced aftereffects of subsequently viewed faces (Ghuman et al., 2010). This cross-category, body-face adaptation suggests a tight coupling of these representations, such that the bodies alone can activate the network underlying face perception.

Cross-category face adaptation has primarily been shown for face identity aftereffects. For instance, Hills et al. (2010) established that face identity aftereffects can be produced by voices and identity-specific semantic information. However, Ryu et al. (2008) suggest that perceived or imagined faces can elicit face identity aftereffects. This complicates the interpretation of other examples of cross-modal face identity adaptation, because it is difficult to rule out the possibility that explicit face imagery could be causing the adaptation. The cross-modal gender adaptation addresses this possibility by reducing specific identity representations that might prompt mental imagery. Other than the body-to-face aftereffect (Ghuman et al., 2010), generally studies of gender adaptation have failed to find cross-modal adaptation. In particular, gender-specific voices do not adapt face perception (Kloth et al., 2010), nor do male and female hands (Kovacs et al., 2006) or gender-specific objects (male and female shoes, lipstick, etc.; Ghuman et al., 2010). These results suggest that the tight, intrinsic conceptual relationship between bodies and faces is what allows for cross-modal perceptual adaptation.

The face inversion effect, wherein accuracy of recognition is reduced and reaction time is slowed when faces are viewed upside down as compared to upright (Yin, 1969; Haxby et al., 1999; Rossion and Gauthier, 2002), is a hallmark of face perception. The face inversion effect is disproportionate in comparison to the physical change in the configuration of the stimulus properties and in comparison to other objects commonly encountered only in the upright orientation (Rossion and Gauthier, 2002). Recent studies suggest that bodies also display a behavioral inversion effect (Reed et al., 2003) analogous to that observed for faces, and the body inversion effect may require the presence of a head and may be mediated by face-selective mechanisms (Brandman and Yovel, 2010). These findings suggest that specialized mechanisms exist in the brain to process upright faces and potentially upright bodies.

Face–face adaptation also shows a degree of orientation dependence. Specifically, gender face adaptation is greater when the orientation of the faces is aligned compared to when the faces are in opposing orientations [i.e., adaptation aftereffects of upright faces (↑F) to ↑F are greater than inverted faces (↓F) to ↑F and aftereffects of ↓F to ↓F are greater than ↑F to ↓F; Rhodes et al. (2004), Watson and Clifford (2006), the full pattern of results is ↓F to ↓F > ↑F to ↓F = ↑F to ↑F > ↓F to ↑F]. Face identity and viewpoint adaptation display a relatively similar pattern of adaptation with regards to inversion, with some quantitative distinctions (Fang et al., 2007; Rhodes et al., 2009; Hills and Lewis, 2012). However, face gender adaptation is reduced, not abolished, when the adaptor and target faces are of opposite orientation (Rhodes et al., 2004; Watson and Clifford, 2006). These results suggest that there are both orientation-dependent and orientation-independent face representations and that face aftereffects reflect adaptation of both.

In the present study, we use these findings as a basis for examining the orientation specificity of the face representations activated and adapted by bodies. Specifically, we compare the magnitude of the body-face adaptation aftereffect for upright bodies (↑B) to ↑F, ↑B to ↓F, inverted bodies (↓B) to ↑F and ↓B to ↓F. We use this paradigm to test between two potential hypotheses: (1) Bodies activate face representations according to the orientation of the body. If this alternative were true, then we would expect the aftereffects for ↑B to ↑F to be greater than ↓B to ↑F and for ↓B to ↓F to be greater than ↑B to ↓F, analogous to face–face adaptation as discussed above (Rhodes et al., 2004; Watson and Clifford, 2006). (2) Bodies activate orientation-independent face representations. If this alternative were true, then we would expect the aftereffects for ↑B to ↑F to be similar to ↓B to ↑F and for ↓B to ↓F to be similar to ↑B to ↓F.

**Figure 1:**
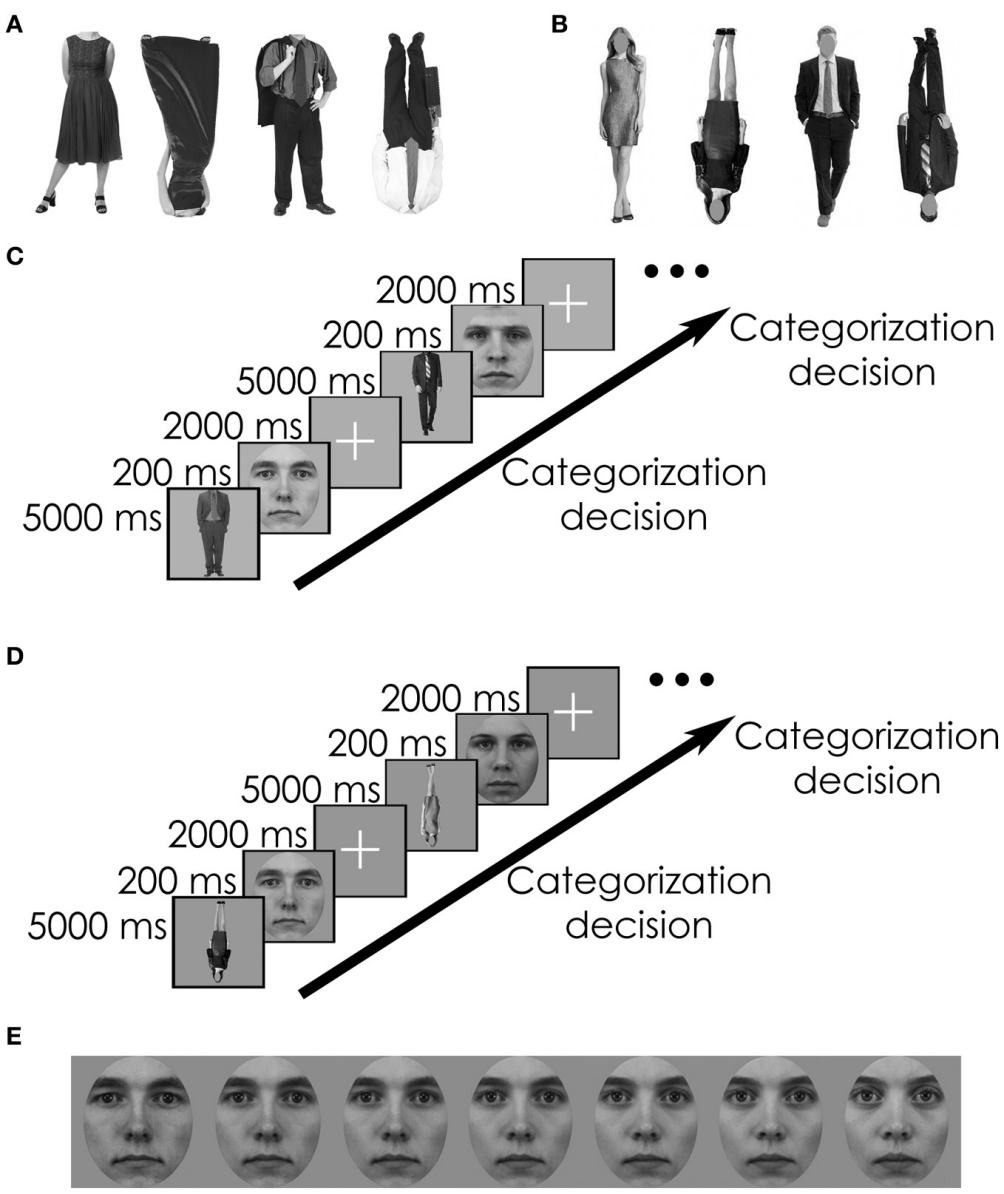
**Examples of stimuli and paradigm. (A)** Gender-specific bodies from the neck down (Experiment 1). **(B)** Gender-specific bodies with faceless heads (Experiment 2). **(C)** Examples of adaptation trial sequences, adjusted from Ghuman et al. (2010) for both experiments. Each trial consisted of an adapting body [male or female, upright or inverted, with (Experiment 1) or without (Experiment 2) head] for 5000 ms. This was followed by a target face (upright or inverted) for 200 ms. Subjects were asked to make a decision about the gender of the face during the presentation of the fixation cross (2000 ms). **(D)** Examples of adaptation trial sequences using inverted adapting body images. Some trials also included inverted faces. **(E)** Examples of 10, 30, 40, 50, 60, 70, and 90% male-to-female face morphs (target stimuli for both experiments).

To test between these hypotheses, we conducted two experiments. In Experiment 1 we examined the orientation dependence of each process by testing the transfer of body-face adaptation between upright and inverted stimuli. The bodies used in this experiment were shown from the neck down, with no visible heads (Figure 1A). Some evidence suggests that the body inversion effect is preserved for bodies with their faces obscured but abolished for bodies without heads (Yovel et al., 2010). Thus, orientation dependence or independence may require the presence of a faceless head. To further explore the role of the presence or absence of a head in body-face interactions, our second experiment replicated the first but involved bodies with obscured faces rather than bodies without heads (Figure 1B).

## Materials and methods

### Subjects

A total of 52 individuals participated in this study. After exclusion due to an inability to distinguish the target faces (responding that the faces came from a single gender on more than 85% of all trials, making it unclear if these subjects were complying with the instructions), there were 25 subjects in Experiment 1 and 21 subjects in Experiment 2. Ages ranged from 18 to 49. All subjects were naïve to the goals of the study. The Institutional Review Board of the University of Pittsburgh approved all procedures and written informed consent was obtained for all subjects.

### Stimuli

Target face stimuli for all experiments were constructed from photographs of 6 male and 6 female frontal-view faces with neutral expressions from the Karolinska Directed Emotional Faces (KDEF; Lundqvist et al., 1998) stimulus set. For each of the 6 male and female face pairs from the KDEF set, male-to-female face morphs were constructed (Figure 1E) using Morpheus Photo Morpher™. Each face image was cropped with a uniform oval that removed all non-facial features. The 10, 30, 40, 50, 60, 70, and 90% morphs were used in these experiments. Adapting body stimuli consisted of photographs of 20 male and 20 female bodies in each experiment. Face pictures in both experiments and body pictures in Experiment 1 were the same as in Ghuman et al. (2010); body pictures in Experiment 2 were collected from the Internet. Adobe Photoshop was used to convert all body and face images to grayscale and to resize the images to best fill a gray square subtending approximately 6.5° of visual angle. Stimuli were presented in the middle of the screen.

### Procedure

The adaptation paradigm was adjusted from Ghuman et al. (2010). For both experiments, each adaptation trial began with subjects viewing an adaptation image [a male or female body, upright or inverted, with (Experiment 1) or without (Experiment 2) a head] for 5 s. Following adaptation, a target face (upright or inverted) was presented for 200 ms followed by a 2000 ms fixation cross in the center of the screen (Figure 1C). Subjects made a two-alternative forced-choice response to classify the face gender as quickly and accurately as possible.

Experiment 1 used images of bodies cropped to remove the head (Figure 1A) as adapting stimuli and male-to-female face morphs as target stimuli. The experiment was divided into four blocks consisting of 78 trials each, with the face and body images' orientations held constant within each block, and faces were never repeated within a block. These blocks were presented in a pseudorandom order, counterbalanced across subjects, so each participant would eventually see every combination of orientations of bodies and faces: upright bodies (↑B) to upright faces (↑F), ↑B to ↓F, ↓B to ↑F, or ↓B to ↓F. Within each block, gender of the body stimuli was also varied pseudorandomly, such that the first half of each block showed bodies of one gender and the second half showed bodies of the other gender. The two halves of each block were separated by a 1-min break. Experiment 2 was identical in structure to Experiment 1, but the adapting body stimuli used here included heads with obscured faces Figures 1B,D. In both experiments, the order of the four conditions was counterbalanced across participants.

### Analysis

Aftereffect magnitude was defined as the percent of faces endorsed as male following adaptation to female bodies minus the percent of faces endorsed as male following adaptation to male bodies. Only face morph levels where subjects gave a particular response less than 80% of the time, averaged across participants and studies, were used to determine aftereffect magnitude and standard error. This is because aftereffects are known to be minimal for unambiguous stimuli. In practice, this meant that the 90 and 10% face morphs were excluded from analysis of aftereffect magnitude. Had these data been included, all significance determinations would have remained unchanged, but the aftereffect magnitude would have been reduced somewhat. The 30, 40, 50, 60, and 70% face morph levels were used for ANOVAs, *F*-tests and *p-values*, analyzed using MATLAB™ and SPSS™. ANOVAs were three-factor tests with two within-subjects factors (“Face” and “Body”) and one between-subjects factor (“Headedness”). The two within-subjects factors were the orientation of the adaptor body and the orientation of the target face, and the between-subjects factor was the presence (or absence) of a head on the body adaptor. The independent variable was the percent endorsed as male in the face categorization decision. In addition, *T*-tests were performed to examine the significance of each of the four within-subject conditions (i.e., orientation of body adaptor and face target).

## Results

Consistent with our previous study (Ghuman et al., 2010), we found that adaptation to a body biased the perception of the gender of the target face in the opposite direction [mean aftereffect across all conditions = 8.9%, *t*_(45)_ = 4.838, *p* < 0.001]. The 2 × 2 × 2 (Face × Body × Headedness) ANOVA revealed no significant main effect of body orientation on aftereffect magnitude [mean aftereffect with upright body = 9.5%, inverted body = 7.2%, *F*_(1, 176)_ = 1.403, *p* = 0.238], and no face x body interaction [*F*_(1, 176)_ = 0.057, *p* = 0.811]. These results suggest that the orientation of the body adaptor does not matter, nor does it interact with the orientation of the face target.

The analysis did reveal a significant main effect of face orientation [mean aftereffect with upright face = 5.8%, inverted face = 10.9%, *F*_(1, 176)_ = 8.276, *p* = 0.005]. These results are consistent with previous reports suggesting that face gender adaptation is larger for inverted target faces than for upright target faces (Rhodes et al., 2004; Watson and Clifford, 2006).

**Figure 2:**
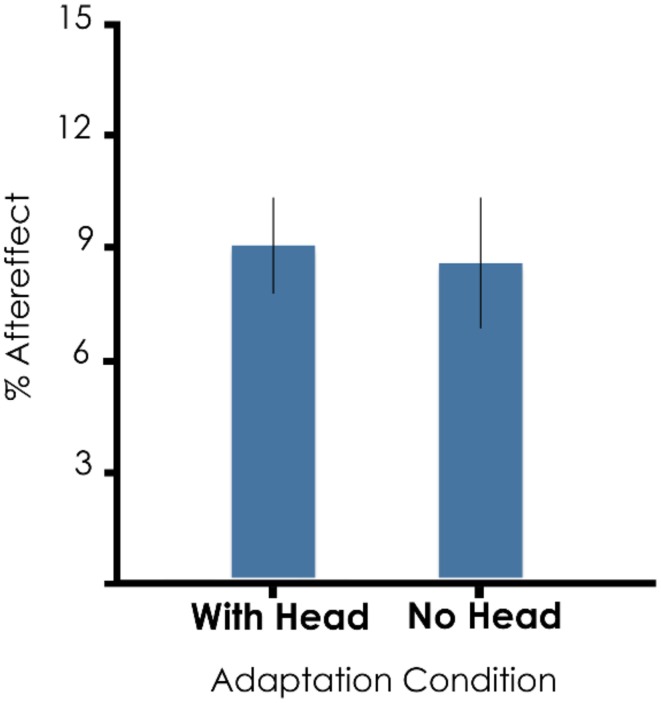
**Aftereffect magnitude across experiments.** Mean and standard error of aftereffects comparing Experiments 1 and 2. The overall mean aftereffect magnitude was 8.9%, calculated as 9.3% for adapting bodies with heads and 8.8% for bodies without heads.

Comparing Experiments 1 and 2, we found no main effect of the presence of a head on aftereffect magnitude [Figure 2; mean aftereffect with head = 9.3%, without head = 8.8%, *F*_(1, 176)_ = 1.057, *p* = 0.305]. Additionally, there were no interactions of face × headedness [*F*_(1, 176)_ = 0.970, *p* = 0.326], body × headedness [*F*_(1, 176)_ = 0.954, *p* = 0.330], or face × body × headedness [*F*_(1, 176)_ = 0.013, *p* = 0.909]. These results indicate that adaptation to bodies with faceless heads and to bodies without heads are similar.

**Figure 3:**
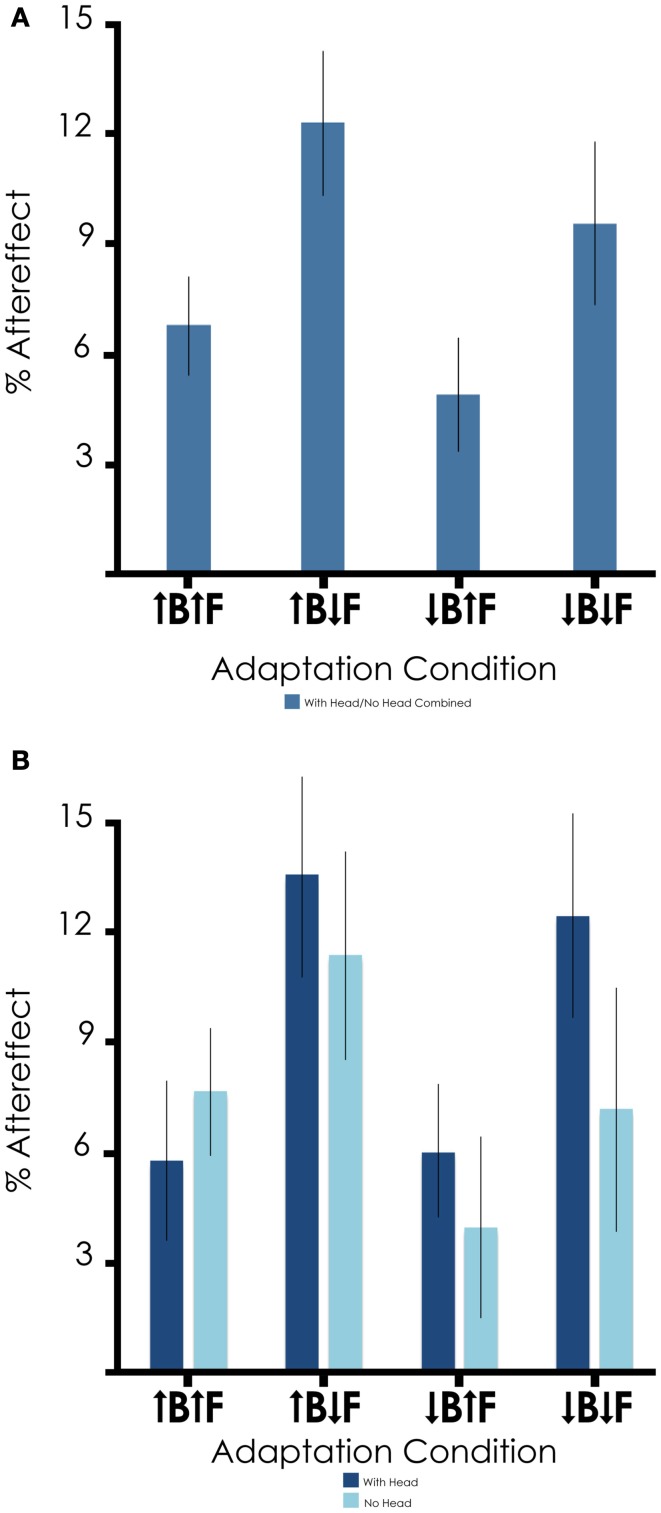
**Summary of aftereffect magnitude across adaptation conditions. (A)** Results are collapsed across Experiments 1 and 2 due to the lack of significance of headedness on the adaptation effects. Aftereffect magnitudes by condition were 6.7% for ↑B to ↑F, 12.3% for ↑B to ↓F, 4.8% for ↓B to ↑F, and 9.5% for ↓B to ↓F, with overall mean aftereffect 8.9%. **(B)** Results from Experiment 1 (with head) and Experiment 2 (no head) shown separately for comparison.

We then examined the results of the four inversion combinations (↑B to ↑F, ↑B to ↓F, ↓B to ↑F, ↓B to ↓F), shown in Figure 3A collapsed across Experiments 1 and 2 due to the lack of significance of headedness on the adaptation effects (see Figure 3B for the data from Experiments 1 and 2 separated out). The magnitude of the aftereffect was 6.7% in the ↑B to ↑F condition [*t*_(45)_ = 4.850; *p* < 0.001], 4.8% in the ↓B to ↑F condition [*t*_(45)_ = 3.055; *p* = 0.004], 12.3% in the ↑B to ↓F condition [*t*_(45)_ = 6.146; *p* < 0.001], and 9.5% in the ↓B to ↓F condition [*t*_(45)_ = 4.249; *p* < 0.001].

## Discussion

The main objective of this study was to investigate the orientation specificity of the face representations activated by bodies. The aftereffect magnitude for ↑B to ↑F was similar to ↓B to ↑F and ↓B to ↓F was similar to ↑B to ↓F. Therefore, these results support the hypothesis that bodies activate orientation-independent face representations. In addition, we also examined the role of inversion in body-face adaptation when the bodies had heads because the results of previous studies suggest that the presence of a head (with the face occluded) is important to face-body interactions and particularly body inversion (Cox et al., 2004; Brandman and Yovel, 2010; Yovel et al., 2010; Brandman and Yovel, 2012). In this case, we found no significant difference in aftereffect magnitude when comparing the results of the two experiments with regard to the presence of a head. Additionally, we did find a main effect of the orientation of the face, such that larger aftereffects were seen for inverted face targets. This result is in line with previous face–face gender adaptation studies (Rhodes et al., 2004; Watson and Clifford, 2006). Finally, we examined each of the individual conditions and found significant aftereffects in all four body and face orientation conditions. Overall, our results replicate previous reports of body-face adaptation (Ghuman et al., 2010) and extend them by suggesting that bodies activate faces in a relatively orientation-independent manner.

Previous studies suggest that upright and inverted faces are encoded by different populations of neurons(e.g., Watson and Clifford, 2006). Several electrophysiological single-unit studies support this assertion, showing neurons responding differently to upright and inverted cartoon faces (Friewald et al., 2009) and whole bodies (Ashbridge et al., 2000). Based on the result that the perception of individual facial features is invariant to inversion (Searcy and Bartlett, 1996; Leder and Bruce, 1998; Freire et al., 2000), one possibility is neuronal populations that encode these features are broadly tuned with respect to orientation, while neurons that encode holistic properties of faces are more narrowly tuned to upright faces (see Maurer et al., 2002; Watson and Clifford, 2006). From this standpoint, the present results would suggest that bodies primarily activate the orientation-independent representations of individual facial features rather than the orientation-dependent holistic representations. Another hypothesis is that, in addition to neuronal populations tuned to facial features that are inversion-invariant, there are neuronal populations tuned to holistic representations of faces that have two different types of orientation tuning. Specifically, there is a population of narrowly tuned neurons responding to upright faces and a population of broadly tuned neurons responding to upright and inverted faces (Sekuler et al., 2004; Watson and Clifford, 2006). From this perspective, our results would indicate that bodies are primarily activating the broadly tuned, orientation-independent neurons encoding holistic aspects of faces.

Two neural regions that are sensitive to static aspects of faces (as opposed to dynamic properties, such as expression and gaze direction) are potential neural loci for body-face adaptation. The first is the occipital face area (OFA), which is primarily selective for individual facial features (Kanwisher et al., 1997; Liu et al., 2010) and responds similarly to upright and inverted faces (Yovel and Kanwisher, 2005; Pitcher et al., 2011). Neuroimaging studies indicate that the OFA and the extrastriate body area (EBA), which is sensitive to body parts (Urgesi et al., 2004; Chan et al., 2010), both respond more strongly to the presence of both a face and a body than to the presence of a face or body alone (Schmalzl et al., 2012). Thus, they may play a role in combining face and body information. While it would be surprising if bodies activated face information at the level of individual features (e.g., more masculine or feminine facial features) rather than at the level of holistic face representations, the relative orientation invariance of the OFA representation makes this possibility consistent with the current data. A second potential neural locus for body-face adaptation is the fusiform face area (FFA), which has orientation-dependent face representations (Yovel and Kanwisher, 2005) and is influenced by lower-level features and configurations (Chan et al., 2010; Yue et al., 2011) as well as more holistic qualities of faces (Liu et al., 2010; Schiltz et al., 2010; Nestor et al., 2011). The close proximity of the FFA to body-selective regions in the fusiform (Peelen and Downing, 2005; Schwarzlose et al., 2005) along with the superadditive response of face and body information in the fusiform (Schmalzl et al., 2012) support the possibility that this area is a neural basis of face-body adaptation. However, the sensitivity of the fusiform gyrus to inversion of faces and bodies (Yovel and Kanwisher, 2005; Brandman and Yovel, 2010) make this hypothesis unlikely. Indeed, the FFA does not seem to be sensitive to high-level aspects of faces, such as identity (Kriegeskorte et al., 2007; Nestor et al., 2011).

Another potential neural locus for body-face adaptation is body-sensitive neural regions. A recent study suggests that the body inversion effect is mediated by face-specific, rather than body-related, mechanisms (Brandman and Yovel, 2010). Specifically, they found that the FFA was sensitive to body inversion, but the extrastriate body area (EBA) was not. Furthermore, the FFA was only sensitive to body inversion when the body included a visible head (with the face occluded), while the EBA was relatively insensitive to the presence or absence of a visible head. Here we demonstrate that body-face adaptation is not sensitive to body inversion and is not sensitive to the presence or absence of a head, paralleling the neural sensitivity of the EBA. This suggests that body-face adaptation may be governed by body-related processing, potentially in the EBA. A recent study demonstrated that the EBA shows a significant ability to discriminate faces (Chan et al., 2010), suggesting that the EBA may represent some face properties. Thus, one potential hypothesis is that bodies adapt face information in the EBA.

A third hypothesis is that neural regions sensitive to joint body-face properties (“person representations”) mediate body-face adaptation. One potential neural locus for person representations and body-face adaptation is the anterior temporal face patch (AT), as it appears important for face individuation and identification (Kriegeskorte et al., 2007; Nestor et al., 2011), responds to whole faces (Nasr and Tootell, 2012), and shows some sensitivity to bodies as well as faces (Pinsk et al., 2009). The orientation sensitivity of AT is difficult to determine as it is downstream of the FFA, and reports of reduced activity in AT for inverted relative to upright faces (Nasr and Tootell, 2012) could be due to the upstream orientation dependence of the FFA rather than orientation sensitivity in AT *per se*. But the evidence that suggests AT is critical for the representation of high-level face information (Kriegeskorte et al., 2007; Nestor et al., 2011) supports the possibility of AT being an important neural locus of face-body adaptation, potentially encoding whole person representations rather than simply face representations. In addition, studies indicate that emotional information from bodies and faces have somewhat overlapping representation (Hadjikhani and de Gelder, 2003; Meeren et al., 2005; Peelen et al., 2010), further emphasizing the relatedness of these representations. If either neural regions sensitive to body or joint body-face properties did underlie body-face adaptation, this would suggest that the cells tuned to this information are involved in the neural representation of the norm-based perceptual face space (Leopold et al., 2001; Webster and MacLeod, 2011).

We found no significant difference between adaptation to bodies with faceless heads and bodies without heads. Previous studies have shown that the presence of a head shape is necessary for many body-face interactions. For example, the body inversion effect has been shown to depend on the presence of a head (Minnebusch et al., 2009; Yovel et al., 2010), the face inversion effect can be induced using bodies with faceless heads (Brandman and Yovel, 2012), and some face and body sensitive regions are activated superadditively in response to bodies and faces (Schmalzl et al., 2012). However, in a visual detection task, the presence of a head did not affect body inversion effects (Stein et al., 2012). Our results seem to indicate that the presence of a faceless head does not modulate body-face adaptation. The reason for this discrepancy between body-face adaptation and the other types of body-face interactions is not entirely clear, but it may be due to the particular face properties being probed. Specifically, many other studies have used facial identity or neural activity as the critical measure of face-body interactions, while ours focused on perceptual adaptation aftereffects of face gender. One potential limitation of the present study is that different body stimuli were used in Experiments 1 and 2. However, the source of the stimuli were similar (websites of clothing retailers; lighting, pose, and orientation of the bodies were similar), so it is unlikely that the lack of a main effect of the presence of a head was driven by the different body pictures used in the two experiments. Our results strongly suggest that bodies with and without visible heads activate and modulate face gender representations equally.

There was a main effect of target face orientation, with larger aftereffects observed for inverted target faces (↑B to ↓F, ↓B to ↓F). While this is consistent with previous studies of face–face adaptation (Rhodes et al., 2004; Watson and Clifford, 2006), the underlying reason is unclear. The simplest explanation is that briefly presented inverted faces are more ambiguous than upright faces, and this ambiguity may result in greater vulnerability to adaptation. Nonetheless, modulation of the aftereffect magnitude by target face orientation demonstrates another similarity between face–face adaptation and body-face adaptation.

A possible explanation for the lack of a significant effect of body orientation is that bodies, regardless of orientation, are specifically activating representations of upright faces rather than activating orientation-independent face representations. Previous studies have shown that upright faces readily adapt the mechanism for perception of inverted faces, eliciting aftereffects of similar magnitude for both upright and inverted face targets (Rhodes et al., 2004; Watson and Clifford, 2006). In contrast, inverted faces cause little adaptation of the mechanism for perception of upright faces (Rhodes et al., 2004; Watson and Clifford, 2006). However, our results show that adapting to bodies produces larger aftereffects for inverted target faces than for upright target faces, which is somewhat inconsistent with the idea that both inverted and upright bodies activate upright faces. While our results do not perfectly align with this idea, it cannot be fully excluded because bodies may activate representations of upright faces that interact with an inverted target face in a way that is unexpected or differs from what occurs when the adaptor is an actual face.

In conclusion, our results confirm that gender adaptation transfers from bodies to faces, and suggest that this effect is invariant to the orientation of the adapting body. The nature of the face representation activated by bodies needs to be clarified by further investigations, such as explorations of retinotopic dependence, size dependence, or other manipulations of visual field properties. Additionally, neuroimaging studies would help elucidate the processing level at which perception of bodies activates face representations. More broadly, body-face adaptation helps demonstrate the overlap between conceptual and perceptual systems, a central tenet of the theory of embodied cognition (Martin, 2007; Barsalou, 2008).

### Conflict of interest statement

The authors declare that the research was conducted in the absence of any commercial or financial relationships that could be construed as a potential conflict of interest.
